# Haplotype-based autoencoders can reduce the dataset dimension and estimate haplotype block effects in different crop species

**DOI:** 10.1186/s12859-025-06323-w

**Published:** 2025-12-02

**Authors:** Philipp Georg Heilmann, Emanuel Grosch, Matthias Frisch, Matthias Herrmann, Steffen Beuch, Vivek Kurra, Martin Mascher, Raz Avni, Klaus Oldach, Ina Röhrs, Anja Hanemann, Raja Ram Mehta, Carsten Reinbrecht, Albrecht Serfling, Andreas Stahl, Marco Stucke, Amine Abbadi, Tobias Kox, Carola Zenke-Philippi

**Affiliations:** 1 Institute of Agronomy and Plant Breeding II, Justus Liebig University, Heinrich-Buff-Ring 26-32, 35392 Giessen, Germany; 2 Research on Agricultural Crops, Federal Research Centre for Cultivated Plants, Institute for Breeding, Julius Kühn Institute, Rudolf-Schick-Platz 3a, 18190 Sanitz, Germany; 3 Saatzucht Granskevitz, Nordsaat Saatzucht GmbH, Granskevitz 3, 18569 Schaprode, Germany; 4 Saatzucht Bauer GmbH & Co. KG, Landshuter Str. 3a, 93083 Obertraubling, Germany; 5 Leibniz Institute of Plant Genetics and Crop Plant Research (IPK) Gatersleben, Corrensstraße 3, 06466 Seeland, Germany; 6 KWS Lochow GmbH, Ferdinand-von-Lochow-Str. 5, 29303 Bergen, Germany; 7 Research & Breeding Dottenfelderhof, Landbauschule Dottenfelderhof e.V., Dottenfelderhof 1, 61118 Bad Vilbel, Germany; 8 Saatzucht Josef Breun GmbH & Co. KG, Amselweg 1, 91074 Herzogenaurach, Germany; 9 Saatzucht Streng-Engelen GmbH & Co. KG, Aspachhof, 97215 Uffenheim, Germany; 10 Institute for Resistance Research and Stress Tolerance, Julius Kühn Institute, Erwin-Baur-Str. 27, 06484 Quedlinburg, Germany; 11 NPZ Innovation GmbH, Hohenlieth-Hof, 24363 Holtsee, Germany

**Keywords:** Plant breeding, Machine learning, Genomic prediction, Neural networks, Autoencoders, Dimensionality reduction, Haplotype blocks

## Abstract

**Background:**

In plant breeding, many studies currently investigate the application of machine learning (ML) to genomic prediction, hoping for an improvement in prediction accuracy compared to standard models like Genomic Best Linear Unbiased Prediction (GBLUP). However, ML algorithms require much higher computational resources. This study aims to reduce the computational requirements and speed up training time by developing a novel autoencoder architecture inspired by haplotype blocks. Our approach incorporates prior knowledge on genetic linkage, inspired by haplotype block building, into the autoencoder architecture, resulting in a new encoded variable per haplotype block. We further modified our model into a semi-supervised version by adding available yield information. We used features extracted from the autoencoder’s block layer as inputs for Random Forest and GBLUP models to predict the yield of hybrid and inbred crops.

**Results:**

Genomic prediction based on the extracted features maintained prediction accuracies equal to using the original marker data, even with a variable reduction of up to 98% and significantly reduced computation time. Prediction accuracy of the supervised component was in some cases equal to and in some lower than the prediction accuracy achieved using GBLUP. Effects estimated for haplotype block variants using our new method showed a high correlation to the blockwise sum of marker effects, which is the current standard approach for haplotype block effects. Correlation between the two block effect estimation approaches was very low for some blocks, which might indicate the incorporation of non-linear effects by the autoencoder.

**Conclusions:**

Our approach introduces a new perspective on processing haplotype blocks for genomic prediction, potentially providing more flexible modelling opportunities without the use of multiple binary dummy variables for each block variant. Additionally, training time for ML models may be significantly reduced by using the reduced feature sets generated using our method. By adding the semi-supervised component, the model is able to estimate values similar to marker effects for each block on yield. In future work, this may provide a new way of quantifying the importance of haplotype blocks for selection and breeding.

## Introduction

With the introduction of marker-based prediction methods [1–3] and the development of high-throughput genotyping, plant breeding has increasingly complemented phenotyping with genotyping, allowing the selection and evaluation of untested genotypes. Methods such as genomic best linear unbiased predictor (GBLUP) provide robust frameworks for predicting the phenotypic values of traits based on genetic data. As field trials are expensive, accurately identifying the most promising genotypes greatly increases their efficiency and maximises the utility gained from the resources available. Therefore, research in plant breeding is heavily focused on developing new methods with higher prediction accuracies. To this day however, the original GBLUP proves to still be competitive and remains a highly reliable standard method that has not yet been consistently outperformed by other methods [4, 5].

Recently, machine learning (ML) has attracted attention in the field of plant breeding. The term ML encompasses a variety of algorithms that typically learn iteratively from training data. These algorithms can theoretically approximate any underlying function that describes the relationship between predictor variables and target variables within a dataset. Due to this inherent ability to also model nonlinear interactions, researchers hoped to find algorithms that would outperform GBLUP (a linear model by design). However, despite initial optimism, no ML approach has yet been shown to outperform traditional methods consistently in genomic prediction. While many studies demonstrate cases in which ML outperforms older approaches, such as GBLUP or Ridge Regression BLUP (RR-BLUP), its success appears to be species-, trait- and dataset-specific [4–10]. As the application of ML in plant breeding is still in its initial stage, this does not mean that ML cannot outperform classical methods, but rather that we need to develop more specific models and architectures for plant breeding [11].

However, ML algorithms have drawbacks that make them harder to train compared to linear mixed models for genomic prediction. While GBLUP generally uses a $n \times n$ genomic relationship matrix based on single nucleotide polymorphism (SNP) markers, where *n* is the number of genotypes, ML methods typically use the raw marker data, resulting in datasets of much higher dimensionality. Combined with the necessity of training many models for hyperparameter tuning and internal cross-validation [12], ML models often require much more training time and strong computer hardware. This slows down the application of ML in scientific studies compared to straightforward linear models. To address these issues, an initial study of 361 German winter wheat genotypes [8] was conducted, where we investigated the use of different sets of input variables, e.g. haplotype blocks and autoencoder features, to improve the training efficiency and increase the prediction accuracy of ML algorithms.

Haplotype blocks are said to have the potential to reduce the dimensionality of the dataset [13] and capture local epistatic effects [14]. To use blocks as features in prediction, every unique combination of marker alleles within a block (i.e. each variant of a block) is encoded using variables that represent the number of copies of each block variant present in the genotype. When we used haplotype-based blocks as input features, we found that the prediction accuracy was comparable to or slightly higher than that of the full set of SNP markers for all algorithms for all traits except one [8]. However, due to the presence of many block variants, the haplotype-based blocks increased the dimension of the dataset rather than decreasing it [15], which further increased computation time.

As a second set of features, the output of the encoding layer of an autoencoder was used. Autoencoders are unsupervised neural networks optimized to encode a set of input features into a lower dimensional space in such a way that it is possible to reconstruct the original data from the encoded state [16]. Although this approach significantly reduced computation time, prediction accuracy declined for all traits and algorithms, presumably because the learned features were less informative than the original SNP markers [8].

Another study [17] proposed an alternative approach using a series of autoencoders instead of one autoencoder with fully connected layers. With this method, a fixed number of adjacent markers were grouped together and used as inputs for small autoencoders, which were then combined in a second stage to generate lower-dimensional representations for genomic prediction. This way, the prediction accuracy remained stable while the data dimension was reduced. The approach of grouping together a fixed number of adjacent markers is somewhat reminiscent of a very similar approach for building haplotype-based blocks using fixed window sizes. However, studies show that a fixed window size approach is usually not the best way of building blocks as prediction accuracy based on those is usually lower compared to predictions using blocks based on linkage disequilibrium (LD) [15, 18].

In this study, we build upon the method proposed in [17] by incorporating prior knowledge of the genetic linkage structure into the autoencoder structure. Instead of grouping markers based on a fixed window size, we define blocks using pairwise LD. Markers within a block are connected locally within the autoencoder, resulting in an architecture that more accurately reflects the underlying genetic structure. This approach enables each haplotype block to be reduced to a single unit in the encoding layer, i.e. a single variable after extraction, leading to a guaranteed dimensionality reduction compared to other haplotype block-based approaches.

Haplotype stacking has been proposed as a possible application for haplotype blocks in breeding [19]. However, this requires the estimation of effects for each block variant. An estimation method for effects and variances of haplotype blocks was first proposed in Voss-Fels et al.[19]. Effects of block variants are defined as the sum of the individual marker effects that belong to the same block. This approach has been adopted in other studies [20, 21]. Adding an output node of a target trait to our autoencoder architecture enables the model to estimate block variant effects by quantifying the contribution of a variant to the trait as the product of the outputs of the block layer and their respective weights. This provides a novel approach to estimating block variant effects beyond summing up individual effects. Additionally, it solves the problem of handling unseen block variants, which frequently occur in breeding programmes as newly generated material or crosses often contain variants that did not exist in the initial population.

The overall goal of this study was to develop an autoencoder-based method that integrates LD-based haplotype blocks into its architecture. By incorporating genetic linkage information into the model, we aimed to improve the efficiency of dimensionality reduction for genomic prediction. Specifically, we aimed to: (1) characterise the features generated by the autoencoder during unsupervised training and evaluate their usefulness for genomic prediction; and (2) explore the potential of semi-supervised learning as an alternative approach for estimating the effects of variants within haplotype blocks.

## Materials and methods

### Materials

We applied our methodology to five datasets in total. Two of these datasets consisted of inbred/double haploid lines while the remaining three consisted of hybrids. All of the datasets contained yield data and genetic markers. We filtered the data, removing genotypes with more than 60% missing markers. Furthermore, we removed markers consisting of more than two alleles, with 10% or more missing values, or with an expected heterozygosity below 5%. If the remaining markers contained missing values, we imputed them using BEAGLE [22].

Dataset **Ra1** was provided by Norddeutsche Pflanzenzucht Hans-Georg Lembke KG and consisted of 746 rapeseed hybrids. After filtering 10,939 markers remained. Dataset **Mz1** was published previously [23] and accessed through the R package ‘sommer 4.2.0’ [24, 25]. It consisted of 1,254 maize hybrids. Genetic data was provided for the parental lines and 28,803 markers remained after filtering. Dataset **Mz2** is based on the data provided for the 2022 maize prediction competition hosted by the ’genomes 2 fields’ project [26] and publicly available via the projects’ website [27]. After preprossessing, it consisted of 4,689 maize hybrids and 267,818 markers for their respective parents. Dataset **Wh1** consisted of wheat lines which were generated from a factorial crossing design within the project MultiResistGS. After preprocessing, 293 lines and 17,221 markers remained. Dataset **Ot1** consisted of oat lines collected during the project FUGE, also from factorial crosses. After preprocessing, 234 lines and 29,457 markers remained. More information on the datasets is provided in Supplementary Table S1 and S2, and Supplementary Fig. S2.

For every dataset, adjusted entry means were either provided for each genotype or were calculated using an appropriate mixed linear model, factoring in environments and local field design. The specific mixed linear model design depended on the dataset.

### Feature engineering

The haplotype blocks used in this study were built using the LD between adjacent markers with a threshold of 0.7 required for a marker to be assigned to a block. Pairwise LD between all markers was based on the $r^2$ measure [28]. We set a tolerance threshold of 1 marker within a block to be below this threshold.

**Fig. 1:**
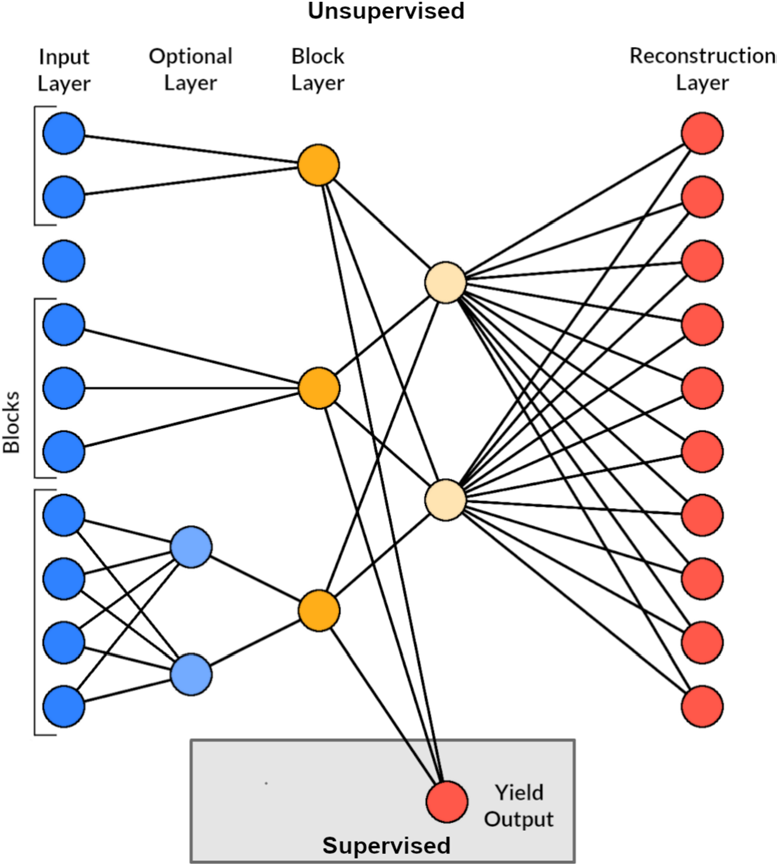
Illustration of the autoencoder architecture

The autoencoder that was used in this study was based on the generated haplotype blocks, as inputs are only connected locally within the borders of these blocks. Figure 1 displays a schematic illustration of an example autoencoder with this architecture. The unsupervised part of the autoencoder was used for feature engineering. It consisted of 4 to 5 layers, depending on the block size. Unsupervised learning refers to models that process input features without regard to any target trait *Y*. The input layer consisted of as many units as there were markers assigned to blocks. Blocks that only consisted of a single marker were not considered blocks and the markers were subsequently removed from the data input. The input layer was locally connected to either all units of an optional hidden layer or to the block layer. If a block contained $\ge 4$ markers, an additional hidden layer was added between the input and the block layer to introduce additional non-linearity and modeling capabilities (“optional layer” in Fig. 1). The optional hidden layer consisted of $\lfloor \frac{n}{2}\rfloor$ units, where *n* was the size of the block. The output of those $\lfloor \frac{n}{2}\rfloor$ units was passed on to the block layer through a leaky ReLU activation function with a negative slope of 0.1. Local connections were limited to markers within the same block, resulting in one single unit per block in the block layer. The block layer was then fully connected to a hidden layer with 1000 units using the same leaky ReLU activation function as before. The hidden layer was connected to the reconstruction layer, which acted as the output of our unsupervised model. This layer returned the reconstructed marker data, which included markers that were previously removed due to a lack of block assignment. As the markers were encoded as -1, 0, and 1 to represent homozygosity for the minor allele, heterozygosity, and homozygosity for the major allele, respectively, the reconstruction layer used a tanh activation function that returned values in the range from -1 to 1.

The autoencoder was trained for 100 epochs. Model parameters were optimized using the Adam optimizer with a learning rate of 0.001 and the mean squared error (MSE) as the loss function. After training, the outputs of the block layer were extracted (first reduction step, AE1). As those were highly correlated, a second dimensionality reduction step (AE2) was added. The block layer output of the first autoencoder was used as the input for a new autoencoder with the same architecture which was then again trained for 100 epochs. Since it was not possible to form LD-based blocks in this scenario, blocks that showed a pairwise correlation >0.7 were grouped together to form ’meta blocks’. This is analogous to [17], where the same procedure was also applied a second time to the already reduced data. Model training was only needed once before the actual cross-validation, since the features could be used in all subsequent cross-validation runs as no phenotypic value was required at this stage.

As an additional reference, principal component analysis (PCA) was used to create principle components as input features, as this is a common and widespread method.

### Genomic prediction

The first model we used to evaluate the influence of dimensionality reduction was a GBLUP. We used two different models for fitting the GBLUP, depending on the type of the crop. For datasets consisting of $i=1,...,n$ homozygous lines we used the model

1 $$\begin{aligned} \textbf{y} = \textbf{1}\beta _0 + \textbf{Zu} + \textbf{e}. \end{aligned}$$

The vector $\textbf{y}$ is the response vector with the phenotypic observations of the $i = 1,...,n$ inbred lines, $\textbf{1}$ is an $n\times 1$ column vector of ones, $\beta _0$ is a fixed intercept, $\textbf{u}$ is a vector of normally distributed random genotypic values with $\text {E}(\textbf{u})=\textbf{0}$ and $\text {cov}(\textbf{u})=\textbf{G}\sigma ^{2}_{A}$, $\textbf{0}$ is an $n \times 1$ column vector of zeroes, and $\textbf{Z}$ is the design matrix for the genotypic values. In models where $\textbf{y}$ contains the adjusted treatment means of a field trial, $\textbf{Z}$ is the identity matrix $\textbf{I}_n$. $\textbf{e}$ is a vector of randomly distributed residuals with $\text {E}(\textbf{e})=\textbf{0}$ and $\text {cov}(\textbf{e})=\textbf{I}_n\sigma ^{2}_{R}$. $\textbf{I}_n$ is an identity matrix and $\sigma ^{2}_{R}$ is the residual variance.

For hybrids, we used a model that included the general combining ability (GCA) and specific combining ability (SCA) as described in [29] and applied in [23]:

2 $$\begin{aligned} \textbf{y} = \textbf{1}\beta _0 + \mathbf {Z_1 u_1} + \mathbf {Z_2 u_2} + \mathbf {Z_s u_s} + \textbf{e}. \end{aligned}$$

The vectors $\textbf{y}$, $\textbf{1}$, $\beta _0$, and $\textbf{e}$ are defined as stated above, this time for the $i=1,...,n$ hybrids. $\mathbf {u_1}$ and $\mathbf {u_2}$ are vectors of normally distributed random GCA effects of parents 1 and 2, respectively, with $\text {E}(\mathbf {u_1})=\text {E}(\mathbf {u_2})=\textbf{0}$, $\text {cov}(\mathbf {u_1})=\mathbf {G_1}\sigma ^{2}_{1}$, and $\text {cov}(\mathbf {u_2})=\mathbf {G_2}\sigma ^{2}_{2}$, $\mathbf {u_s}$ is a vector of normally distributed random SCA effects associated with the specific combinations of parents 1 and 2, with $\text {E}(\mathbf {u_s})=\textbf{0}$ and $\text {cov}(\mathbf {u_s})=\mathbf {G_s}\sigma ^{2}_{s}$, and the design matrices $\mathbf {Z_1}$, $\mathbf {Z_2}$, and $\mathbf {Z_s}$ link the observations in $\textbf{y}$ to the corresponding GCA and SCA effects.

The genomic relationship matrices $\textbf{G}$, $\mathbf {G_1}$ and $\mathbf {G_2}$ were calculated using method I of VanRaden [30]. $\mathbf {G_s}$ is defined as the Kronecker product $\mathbf {G_1} \otimes \mathbf {G_2}$. The variance components were estimated by restricted maximum likelihood (REML).

As an additional reference to evaluate the influence of dimensionality reduction in the context of ML, we used Random Forests [31]. We trained Random Forest models based on 20 different sets of hyperparameters, generated using a maximum entropy grid [32, 33]. Each set of hyperparameters was evaluated using a 5-fold cross-validation based on the training set. We tuned the number of trees ([100, 1000]), the column sampling rate ($[\frac{ncol}{100},\frac{ncol}{3}]$) and the minimum observations required for an additional split ([1, 20]). We then created a stacked ensemble based on the models trained for every hyperparameter combination [34]. We used the LASSO algorithm [35] as the super learner for the new model.

### Estimation of haplotype block effects

Baseline block effects were calculated using the sum of individual marker effects belonging to the same block [19].

To estimate block effects using the autoencoder, the block layer was directly connected to an additional unit that returned a prediction for yield (“yield output” in Fig. 1), which added a supervised part to the autoencoder. While both supervised and unsupervised parts of the model were optimized simultaneously, the supervised part was only optimised on the basis of observations included in the training set while the unsupervised part of the model was optimised on the full data set. This combination of supervised and unsupervised, or labelled and unlabelled, data is known as semi-supervised learning [36].

For the semi-supervised form, the autoencoder used a custom loss function

3 $$\begin{aligned} \text {MSE}(X,\hat{X}) - \text {cor}(y,\hat{y}) + ( \text {MSE}(y,\hat{y})+\lambda ||w_Z||^2) \end{aligned}$$

where *X* represents the true markers and $\hat{X}$ the reconstructed markers, *y* represents the true yield and $\hat{y}$ the predicted yield, and $\lambda$ is the ridge parameter of the squared $\ell _2$-norm of the parameters of the block layer $w_Z$. The term $\text {MSE}(X,\hat{X})$ is the loss function for the unsupervised part of the autoencoder. It represents the difference between the output of the autoencoder, which are the reconstructed markers, and the true markers that were used as inputs. By minimizing this error, the model learns to form a meaningful latent representation of the marker data in the block layer. Minimizing the negative correlation between the observed and predicted yield is equal to maximizing the same correlation. It is important that the rankings of observed and predicted phenotypes match, especially for the best observed genotypes, as these are the ones selected in a breeding program. Since the correlation is scale-free, the term $( \text {MSE}(y,\hat{y})+\lambda ||w_Z||^2)$ is introduced which minimizes the MSE between predicted and observed trait values. This ensures that predictions are on the same scale as the observed values. Similar to the ridge parameter in methods like RR-BLUP, $\lambda ||w_Z||^2$ leads to a shrinkage of the parameters connecting the block layer and the supervised part. This shrinks the parameters of blocks with lesser importance for the final prediction. In addition, the constraint encourages the model to rely more on the shared block representation, thereby stabilizing training and reducing the risk of overfitting in the supervised part. We set $\lambda$ to 0.001 for every dataset.

### Evaluation

A Mantel test [37] was used to assess the autoencoders ability to capture the genomic information in a lower dimensional space. A Mantel test compares the similarity between the distance matrices of the target matrices, in our case the genomic relationship matrices generated from the full SNP data and both reduced feature sets. Genomic relationship matrices were calculated according to method I of VanRaden [30].

To assess the suitability of our data for genomic prediction, we used GBLUP and RF based on the full marker data as the reference models. The features from the first and second reduction step were rescaled to be between -1 and 1 to compute the genomic relationship matrices used in GBLUP as this format was required by the software. The RF model used untransformed features.

For cross-validation, 100 training and test sets based on 80%/20% of the data were created and tested with each combination of method (GBLUP and RF) and feature set (full SNPs, reduction step 1 and reduction step 2). Additionally, 100 training and test sets were created for data sets of hybrid crops (Ra1, Mz1, Mz2) for which the test sets only consisted of T0 hybrids, i.e. hybrids where both parental lines did not appear in the training set. The prediction accuracy of a model was defined as the correlation between the observed and predicted yield. Prediction accuracy and computation time were recorded for every cross-validation run.

To explore the potential of semi-supervised autoencoders for genomic prediction and variant effect estimation, the prediction accuracy of the semi-supervised version of the autoencoder was compared to the standard GBLUP model using the same initial 100 training and test sets. Additionally, the haplotype block variant effects derived from the autoencoder were compared to the sum of the single marker effects belonging to the same block. The marker effects were derived from the GBLUP. As GBLUP and RR-BLUP are mathematically equivalent [38, 39], it is possible to transform the random effects of the genotypes into marker effects.

### Software

Autoencoders were computed using Python 3.12 and PyTorch 2.5.1 [40] using an NVIDIA®Quadro RTX™4000 GPU. All other calculations were done using R 4.3.3 [41] on two Intel®Xeon®Platinum 8276 CPU. Marker filtering and haplotype block building was done using routines which we programmed in the C and R programming languages. Mantel test was done using the R package ‘vegan’ [42]. Genomic relationship matrices and GBLUP were calculated using ‘sommer 4.2.0’ [24, 25], for RF we used the ‘tidymodels’ framework [43] with ‘ranger 0.16.0’ [44]. Due to its large size, GBLUP for dataset Mz2 was calculated using ASReml 4.2.0.267 [45].

## Results

### Dimensionality reduction for genomic prediction

The magnitude of the dimension reduction is shown in Table 1. In general, the dimension of the features was reduced to 9-15% of the original dimension, where all datasets fell in the range of 13-14% with the exception of Ot1 ($\sim$10%). After the second reduction step, only around 1-3% of the original dimensionality remained.

**Table 1:** Absolute and relative dimension of the full set of SNPs (SNP), the output of the first autoencoder (AE1) and the second reduction step (AE2) for all datasets. Additionally, amounts of markers with no block assignment are listed

	SNP	AE1	AE2	Unassigned SNPs
Ra1	10939 (100%)	1458 (13.33%)	177 (1.62%)	1472
Mz1	28803 (100%)	4140 (14.37%)	534 (1.85%)	14061
Mz2	267818 (100%)	34643 (12.94%)	3819 (1.43%)	106500
Wh1	17221 (100%)	2353 (13.66%)	427 (2.48%)	5806
Ot1	29457 (100%)	2890 ( 9.81%)	432 (1.47%)	3090

A Mantel test was used to compare the similarity between all the genomic relationship matrices (Table 2). All genomic relationship matrices had a significant *r*-value (*p*-value < 0.001) which indicated a high correlation between all tested matrices. The Mantel test for comparing the full set of SNPs to the first autoencoder features showed high variability between the datasets. While the two maize datasetes showed very high similarity (values above 0.9), the oat dataset showed much lower similarity (around 0.5). The same pattern could be observed for the comparison of the SNPs to the features of the second autoencoder. The *r*-values generally showed the same tendencies as the previous test, with only small differences. Features AE1 and AE2 seemed to result in very similar genomic relationship matrices as the *r*-values were very high for all datasets (>0.9).

**Table 2:** Mantel test of similarity between genomic relationship matrices derived from the full set of SNPs (SNP), the output of the first autoencoder (AE1) and the second reduction step (AE2) for all datasets. Results are provided in the form: *r*-value (*p*-value). The *r*-value stands for the correlation and ranges from -1 to 1 where 1 is perfect correlation

	SNP vs AE1	SNP vs AE2	AE1 vs AE2
Ra1	0.7559 (<0.001)	0.7176 (<0.001)	0.9669 (<0.001)
Mz1	0.9988 (<0.001)	0.9834 (<0.001)	0.9886 (<0.001)
Mz2	0.9499 (<0.001)	0.9348 (<0.001)	0.9855 (<0.001)
Wh1	0.7907 (<0.001)	0.8159 (<0.001)	0.9807 (<0.001)
Ot1	0.5322 (<0.001)	0.5392 (<0.001)	0.9419 (<0.001)

**Fig. 2:**
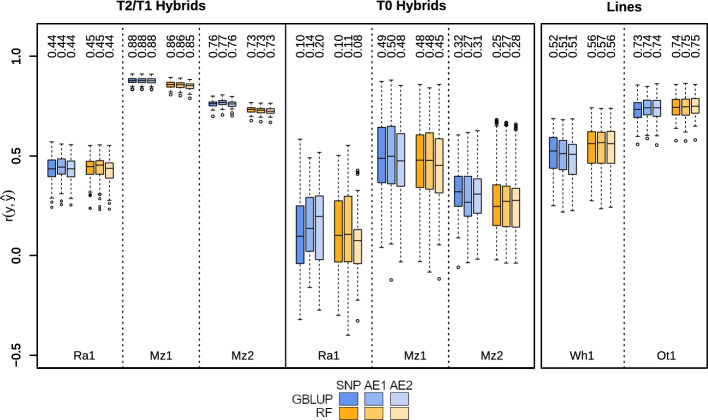
Prediction accuracy of all cross-validation runs. Blue boxplots indicate GBLUP and orange indicate RF, wheares the reduced saturation indicates reduced feature sets. Numbers above the boxplots indicate the median prediction accuracy. Left shows T2/T1 hybrids, i.e. the 100 regular cross-validation runs where either one (T1) or both (T2) parental lines of the hybrids in the test set were available in the training set. The center shows 100 cross-validation runs where parental lines of hybrids in the test set were removed from the training set (T0). Plot on the right shows prediction accuracy for the cross-validation of the line datasets

Results of the cross-validation for hybrids are shown in Fig. 2. For hybrids, the reduction in the data dimensionality did not lead to a decrease in prediction accuracy. In the cross-validation scenario where parents of the hybrids in the test set also appeared in the training set (T1/T2 hybrids), prediction accuracy was equivalent for different feature sets within algorithms. In the cases of Ra1 and Mz1, prediction accuracy was also equivalent between algorithms while RF performed slightly worse for dataset Mz2. In the case of the T0-scenario, prediction accuracies within algorithms showed slightly more variability between feature sets. The changes in the prediction accuracy were still very minor, with the only exception being GBLUP for Ra1, where prediction accuracy improved from 0.1 to 0.14 and 0.2 with the first and second reduction step. This was not observed for RF for the same dataset. Similar results were observed for the inbred datasets (Fig. 2). For lines, the prediction accuracy was equal between the two algorithms and across feature sets. Prediction accuracies were generally higher than when principle components were used as inputs (Figure S3).

The GBLUP was much faster to compute and computation time was mostly unaffected by the change in the data dimension (Table 3). The average computation time for one RF run, including grid search, was drastically reduced in both steps. While training the RF models with the full set of SNPs took several minutes minimum for all algorithms, even up to more than two hours per run in the case of Mz2, computation time of three of the datasets (Ra1, Ot1, Wh1) was reduced to under one minute after the first reduction step, and Mz1 requiring under one minute after the second reduction step. For dataset Mz2, a reduction from more than two hours to 30 to 4 min after the first and second reduction steps could be observed. Computation time after the second reduction step was even faster than the GBLUP for the same dataset with the full SNP data.

**Table 3:** Mean computation time in minutes. GBLUP and RF refer to the methods used. SNP, AE1 and AE2 refer to the feature set used with each method and represent the full set of SNP markers (SNP), the output of the first autoencoder (AE1) and the second reduction step (AE2)

	GBLUP SNP	GBLUP AE1	GBLUP AE2	RF SNP	RF AE1	RF AE2
Ra1	0.23	0.08	0.09	4.19	0.94	0.20
Mz1	0.10	0.49	0.53	21.86	2.84	0.93
Mz2	7.37	7.22	9.77	162.49	34.32	4.35
Wh1	>0.01	>0.01	>0.01	2.64	0.57	0.18
Ot1	>0.01	>0.01	>0.01	3.98	0.79	0.21

### Semi-supervised estimation of block effects

**Fig. 3:**
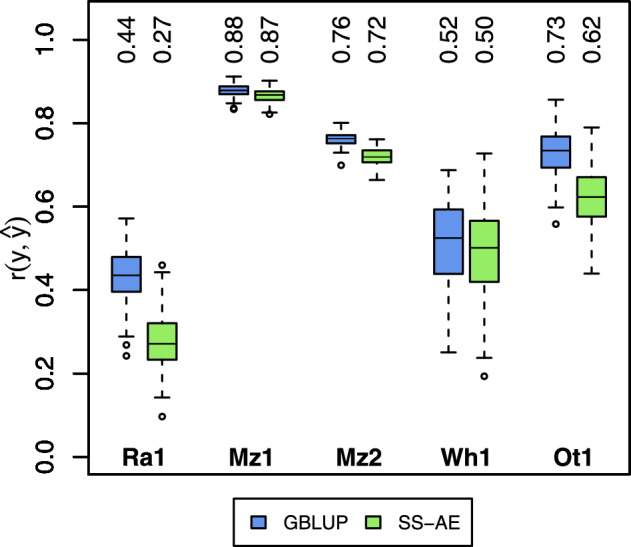
Prediction accuracy of all cross-validation runs for all datasets. Green boxplots indicate the semi-supervised autoencoder (SS-AE), blue boxplots indicate GBLUP. Numbers above the boxplots indicate the medians of the prediction accuracy. For hybrids, training and test sets were identical to the T2/T1 scenario

We observed varying differences in the prediction accuracy between the semi-supervised autoencoder and the GBLUP (Fig. 3). Generally, the semi-supervised autoencoder performed worse than the GBLUP. While prediction accuracy for both methods was equal for datasets Mz1 and, to some degree, Mz2 and Wh1, the prediction accuracy of the autoencoder was lower compared to GBLUP for datasets Ra1 and Ot1.

**Fig. 4:**
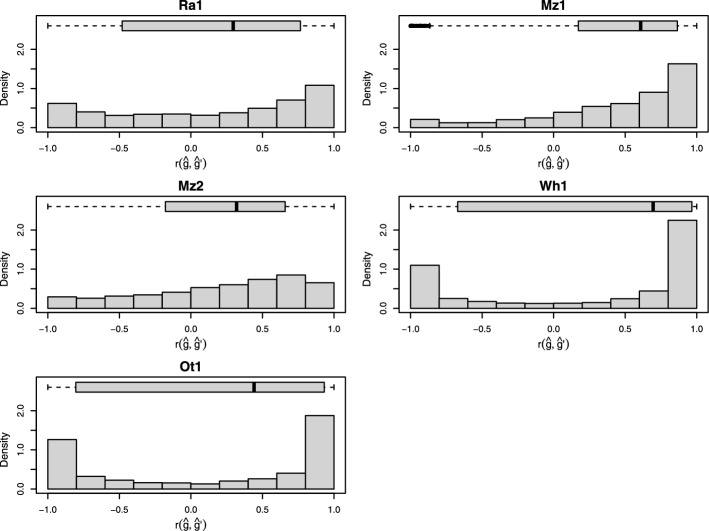
Histograms and boxplots showing the distribution of correlation between effects of encoded blocks ($\hat{g}$) and summed up marker effects ($\hat{g}^\prime$). For each block individually, correlation was recorded between autoencoder-based variant effects and marker effects summed up for every variant

We compared the haploblock variant effects estimated by the semi-supervised autoencoder to the marker effects estimated by GBLUP, summed up for every block. Figure 4 shows the distribution of the correlation between those variant effects for each block. The figure is exemplary and based on the effects estimated for the first cross-validation run for every dataset. The overall tendency was towards a positive correlation between both methods. However, different patterns in the distribution of the correlations could be observed. While Ra1 showed a tendency towards having more observations in the tails of the distribution, it also showed some similarity to a left-skewed distribution. The distributions for Mz1 and Mz2 showed a clear left-skewed distribution with relatively few of the observed correlations falling below 0 and with each median correlation above 0.5. For the line datasets Wh1 and Ot1, the distribution showed a u-shape with most of the blocks falling into either a nearly perfect positive or negative correlation, with a stronger tendency towards a positive correlation.

**Fig. 5:**
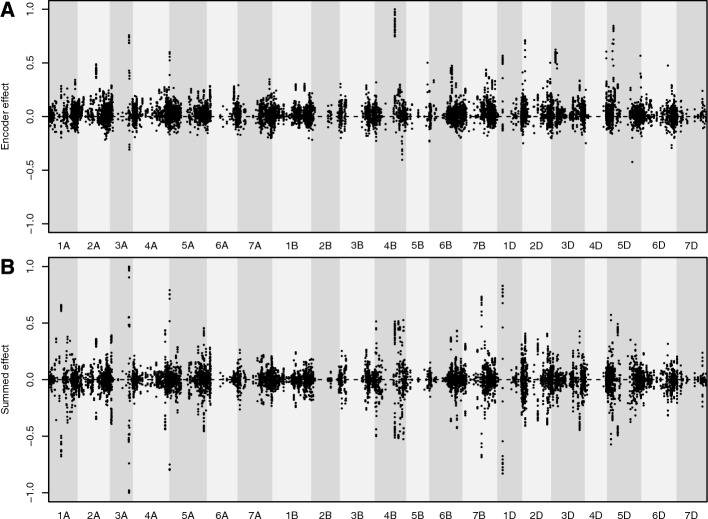
Comparison of effects estimated for each haplotype block variant using the semisupervised autoencoder **A** to the block-wise sum of effects estimated using a GBLUP **B**. Estimation is based on the first cross-validation run for dataset Wh1. Differently highlighted areas indicate different chromosomes. Position on the x-axis indicates physical distance between blocks. The y-axis was scaled for both methods individually through dividing all effects by the largest effect of the respective method, thus scaling all effects from -1 to 1

A direct comparison of block variant effects based on the autoencoder and the block-wise sum of the marker effects is shown in Fig. 5. The observed values were scaled for both approaches due to the absence of an intercept in the autoencoder, resulting in larger effect sizes compared to those generated using GBLUP. The overall trends in effects were comparable, but the distribution of block variant effects was more symmetrical around 0 for single markers. For the encoded effects, there was a stronger tendency toward positive effects (above 0), while fewer negative effects, especially strong negative effects, were observed. Effects from the autoencoder showed some extreme positive values, relative to which most other effects appeared relatively small. Marker-based effects were more evenly distributed across all blocks, with fewer extreme values.

## Discussion

### Autoencoders for dimensionality reduction

In this study, we developed a novel autoencoder architecture based on the concept of haplotype-based blocks to compress high dimensional genetic data while preserving its core information content. Our first goal was to assess how much relevant information is retained within the encoded features extracted from the autoencoder and to determine their usefulness as inputs for prediction models. The Mantel test on the different feature sets showed a high similarity between the genomic relationship matrices generated with those features (Table 2). While all genomic relationship matrices were significantly correlated with each other, the *r*-value between full and reduced data was comparatively low for dataset Ot1. For the maize datasets, the similarity was nearly perfect between full and reduced data. We conclude from this that the majority of genetic relationships contained within the marker data are retained during dimensionality reduction. Additionally, the genomic relationship matrices based on the first and second reduction steps were highly similar in all cases. This implies that reducing the already compressed data further does not result in the loss of any more information regarding the relationship structure in our data. This is reaffirmed by the consistency in the observed prediction accuracy for all datasets and algorithms. Compared to a widespread dimensionality reduction method, PCA, prediction accuracies of RF based on the autoencoder features were also higher (Figure S3). In all cases, prediction accuracy remained largely unaffected by the change in the dimensionality (Fig. 2). Through the dimensionality reduction of approximately 85 to 90% in the initial step and up to 98% in the second step (Table 1), computation time for ML algorithms was drastically reduced (Table 3). Computation time for GBLUP was unaffected as the dimension of the genomic relationship matrices depends only on the number of genotypes tested. Fluctuations in computation time may be related to a higher workload on the server side or other factors beyond the scope of this study.

In summary, we generated features in which each haplotype block is represented by a single variable, and the variables representing the blocks contain most, if not all, of the information from the full marker data. As our model guarantees dimension reduction, it overcomes the typical problem of an increased dataset dimension encountered with haplotype blocks [15]. This may help to avoid the potential problem of decreased prediction accuracy due to multicollinearity when there are many block variants [46]. We conclude that locally connecting markers belonging to the same blocks based on flanking LD in an autoencoder is an effective way to reduce the dimensionality of genetic data. Our method therefore solves the problems faced in an earlier study [8], where autoencoders did not seem to be useful when using a standard architecture based on fully connected layers, as the prediction accuracy declined drastically. By reducing the number of features in the dataset training models for RF and other ML algorithms takes much less time and uses fewer computational resources. This can be advantageous in many situations, such as when computation time or model size are critical factors or related to costs (e.g. if rented cloud computing space is used or local space is limited), or during hyperparameter tuning, which is an integral part of ML [12]. A smaller model could be used to quickly find optimal regions in a very large hyperparameter space or create large ensembles of models. This is important as ensembles of models often perform better than individual models [7, 47, 48], especially when the ensemble is made up of diverse models [49]. The additional cost of training the unsupervised autoencoder is comparatively low as it only has to be trained once and does not require phenotypic measurements. Similar to the popular ML approach of transfer learning [50], a fully trained model can be stored and fine-tuned for each new cycle of a breeding program using newly generated data or for modeling new traits closely related to the original task in its semi-supervised form. This process allows the model to continually improve and adapt to the evolving germplasm within the breeding program.

Autoencoders are rarely used for genomic prediction in plant breeding. Existing studies focus on 2- and 3-dimensional [51, 52] or hyperspectral data [53, 54]. Tross et al. [54] used autoencoders to reduce the dimension of hyperspectral data, where some autoencoder features also showed similarity to known genes influencing leaf phenotypes. To our knowledge, the study that comes closest to ours is Islam et al [17]. Our findings regarding prediction accuracy are in accordance with the findings in their [17] study. However, our method has some key differences. The most notable is that we used a single autoencoder to train the full model, whereas Islam et al [17] used a series of small autoencoders. The equivalent to this would be training an individual autoencoder for each block, which our method can relatively easy be adapted to. However, as we have fully connected layers in the decoder part and also include the markers without block assignment in the output layer, we argue that having a single autoencoder comes with the advantage of the model learning the interconnectedness between blocks and all markers. This can be important as one possible reason for markers not being assigned to a block is that their physical position is not determined correctly and therefore actual existing blocks are broken up. We assume that in our case, a unit in the block layer would also be a good predictor for markers with wrong physical positioning and this information is therefore also accounted for in the model. Secondly, Islam et al. [17] chose to one-hot encode markers, therefore quadrupling the amount of input variables. The advantage is that their approach is not limited to biallelic SNP markers. However, as most markers are biallelic, we believe that this increases computation time unnecessarily in most cases.

### Haplotype block effects

Our second goal was to enhance our model to create a novel approach of estimating the effects of haplotype block variants that goes beyond simply adding up the local effects of markers. For this, we extended our model to include a yield output layer which was used only for those training samples that were in the training set, while the unsupervised part was using all of the data. This concept is called semi-supervised learning [36] and to our knowledge, this approach has not ben utilized in plant breeding before. The advantage of semi-supervised learning is that it uses all of the data in the model. In supervised models, genetic data without phenotypic data is usually excluded completely during model training. This is important because it is often hypothesised that datasets in plant breeding might be too small for ML to work in general [4, 11, 55]. By maximising data usage, breeders can get the most out of the available data.

Comparing GBLUP and semi-supervised autoencoders, the results showed that GBLUP was performing better, albeit by a smaller (Mz1, Mz2, Wh1) or larger (Ra1, Ot1) margin (Fig. 3). One possible explanation is that we used the same approach for every dataset. Typically, the number of epochs, the learning rate, and architectural details such as the size of the hidden layer before the output layer are adapted for each dataset. In our study we prioritised creating a general framework and prove the feasibility of a novel method of estimating haplotype block effects instead of working on fine-tuning the model for every dataset as this would have unnecessarily increased the complexity of the methodology. It is reasonable to assume that prediction accuracy would further improve with dataset-specific parameters.

Some authors argue that Neural Networks may not be suitable for estimating marker effects when used for genomic prediction [56]. However, by incorporating prior knowledge about genetic linkage into our model architecture and a direct connection between block and yield output layer, we ensure that the effects are, by design, in the same unit as the target variable. The effects derived from the autoencoders and the block-wise sums of marker effects [19] were often highly correlated (Fig. 4), especially for datasets Wh1 and Ot1. This agreement between the methods confirms that the autoencoder effects are plausible and meaningful. However, since there were also many cases where the correlation was lower, it appears that the autoencoder identified alternative regions with important contribution to estimating the final yield. This may indicate areas where non-additive effects may play a role. It has been shown that haplotype blocks capture local epistatic effects to some degree [14] although with varying success [57]. As we optimize our model towards two objectives (yield and the reconstructed marker data) and pass the inputs through multiple layers with non-linear activation functions, our model is designed in a way that could help capture local epistatic effects. While this was not directly tested here, future work could explore whether the model can also capture more complex non-linear interactions beyond local epistasis, such as global epistatic effects. Training on a combination of labelled and unlabelled data may also act as a form of regularisation, leading to better model generalisation [58]. Our approach therefore provides an alternative method of estimating the effects of haplotype blocks directly on the variant level instead of relying on estimating effects for markers first. This could be used for proposed methods like “haplotype stacking”, where the goal is to bring favourable combinations of blocks together [19, 21], as this is currently relying on block-wise sums of marker effects.

### Limitations

A current limitation of our model is that, due to the use of a (-1, 0, 1) marker encoding, some haplotype block variants are effectively lost for hybrid datasets. This encoding does not take into account on which chromosome an allele is positioned. The following example (Figure S1) illustrates the problem: The cross between two homozygous parental lines where one has the block variant CCAC and the other CAAA would result in a block variant encoded as (-1, 0, 1, 0). Another cross between two different homozygous parents with the variants CAAC and CCAA would equally result in a hybrid with marker data (-1, 0, 1, 0). As a result, our models assigns the same effect to some block variants that are functionally different. This problem does not occur in the case when lines are predicted as they are homozygous.

While this is a constraint when estimating block effects for hybrids, this problem can be circumvented in future studies. A potential solution would be to provide a tuple of the two alleles (i.e. two variables) instead of a single binary variable. This would effectively double the size of the input and unsupervised output layers, while the principle of the architecture remains unchanged. The idea of representing SNPs with two codes, typically one for additive and one for dominance effects, has long been used in quantitative genetics and genomic prediction [30]. Recent implementations in efficient, multi-locus mixed models [59, 60] demonstrate the continued relevance of this approach for GWAS. A similar extension based on design or genomic relationship matrices could also be applied in our framework by using two separate matrices to represent the two parental haplotypes in a block, thereby addressing the problem of ambiguous encodings. Adjusting our method would be required if a diverse set of genotypes with a high rate of heterozygosity is used. Any aspect related to our first objective was not affected as the primary goal was dimensionality reduction while maintaining a stable prediction accuracy. Therefore, the actual values of the features are not interpreted.

## Conclusion

Our method overcomes the limitations of autoencoders that were encountered in previous studies, where prediction accuracy decreased when features generated using an autoencoder where used as inputs in another model. The novel haplotype-based autoencoder successfully compresses high-dimensional genetic data while preserving genetic relationships, as shown by the high similarity in relationship information and consistent prediction accuracy compared to the standard GBLUP model. This dimensionality reduction significantly decreases computation time for ML algorithms, making them advantageous for scenarios where computational efficiency is important or extensive hyperparameter tuning is required. Additionally, the semi-supervised learning approach offers a new way of estimating the effects of haplotype blocks that goes beyond simply adding up the individual marker effects for each block.

## Additional file

Supplementary file 1.

## Data Availability

The datasets Ot1 and Wh1 were originally generated within the research projects SEEH, FUGE, and MultiResistGS. Datasets Ot1 and Wh1, together with the R and Python scripts used in this study, are available in the GitHub repository: https://github.com/PGHeilmann/Haploencoder. Dataset Mz1 is accessible through the R package "sommer" and was originally published in Technow et al. (2014). The dataset Mz2 was obtained from the Genomes to Fields (G2F) initiative and is publicly available through the official project website: https://www.genomes2fields.org/resources. Dataset Ra1 was provided by NPZ Innovation GmbH for internal use and restrictions apply to the availability of these data.
