# Supplementary material for: Haplotype-based autoencoders can reduce the dataset dimension and estimate haplotype block effects in different crop species
Source: BMC Bioinformatics. 2025 Dec 2;26:289. doi: 10.1186/s12859-025-06323-w (PMC12670737; doi:10.1186/s12859-025-06323-w)
Supplement: Supplementary file 1 [file 12859_2025_6323_MOESM1_ESM.pdf]

Supplementary File 1: Haplotype-based Autoencoders Can Reduce the Dataset Dimension and Estimate Haplotype Block Effects in Different Crop Species

Supplementary Tables and Figures

Table S1 Genotypes generated for each cross between parental lines for dataset **Ot1**. Parent 1 was used as a resistance donor for *Fusarium spec.*. Parent 2 was a genotype with superior potential for yield. The remaining genotypes in the dataset were the parental lines themselves.

	Parent1					
Parent 2	Jaak	Keely	Odal	PGL228	Puhti	Zorro
	n					
Apollon	5		3		3	8
Armani		12			6	2
DCAs2PGL-253inH817					21	
Delfin	8	7	2		12	
HSH_PanFläti				19	21	
Max		7	15		9	18
Sy12/1					22	
Sy3512				4		
Symphony	2		7		4	7

Table S2 Genotypes generated for each cross between parental lines for Dataset **Wh1**. Parent 1 was a resistance donor for either *Zymoseptoria tritici*, *Pyrenophora tritici repentis*, or *Fusarium spec.*. Parent 2 was an elite breeding line.

	Parent1				
Parent 2	20812.2.2	XX41	XX45	Stb19/Lorikeet	HTRI1410
	n				
Asory	15			16	38
Informer	1			16	9
Kamerad	29			21	1
LG Initial	23	9	4	41	11
LG Mocca	22			20	17

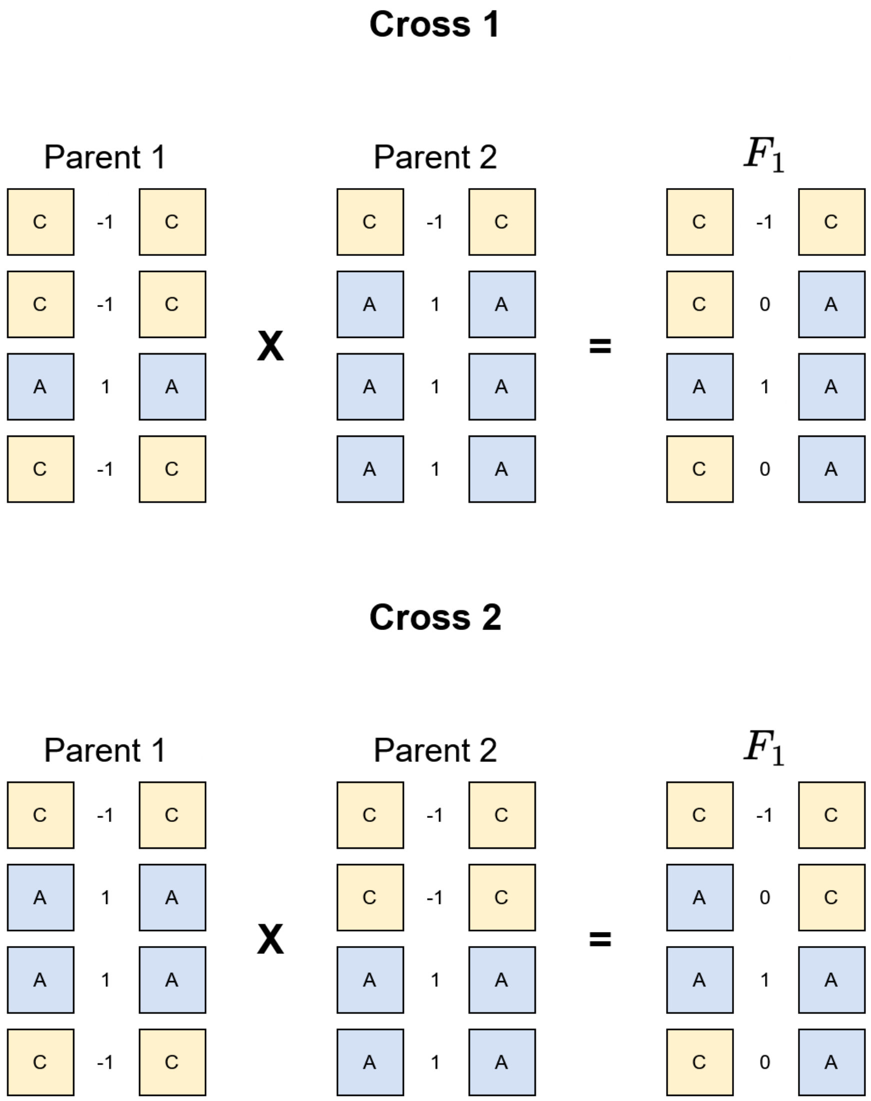

Fig. S1 Illustration showing the encoding problem encountered with hybrids. An example of haplotype block variants containing different alleles and their respective encodings of two genotypes resulting from crosses between different parents is displayed. Although the two genotypes have identical encodings, the block variants differ. This illustrates the issue that different block variants may be treated as the same variant using the current encoding in our model.

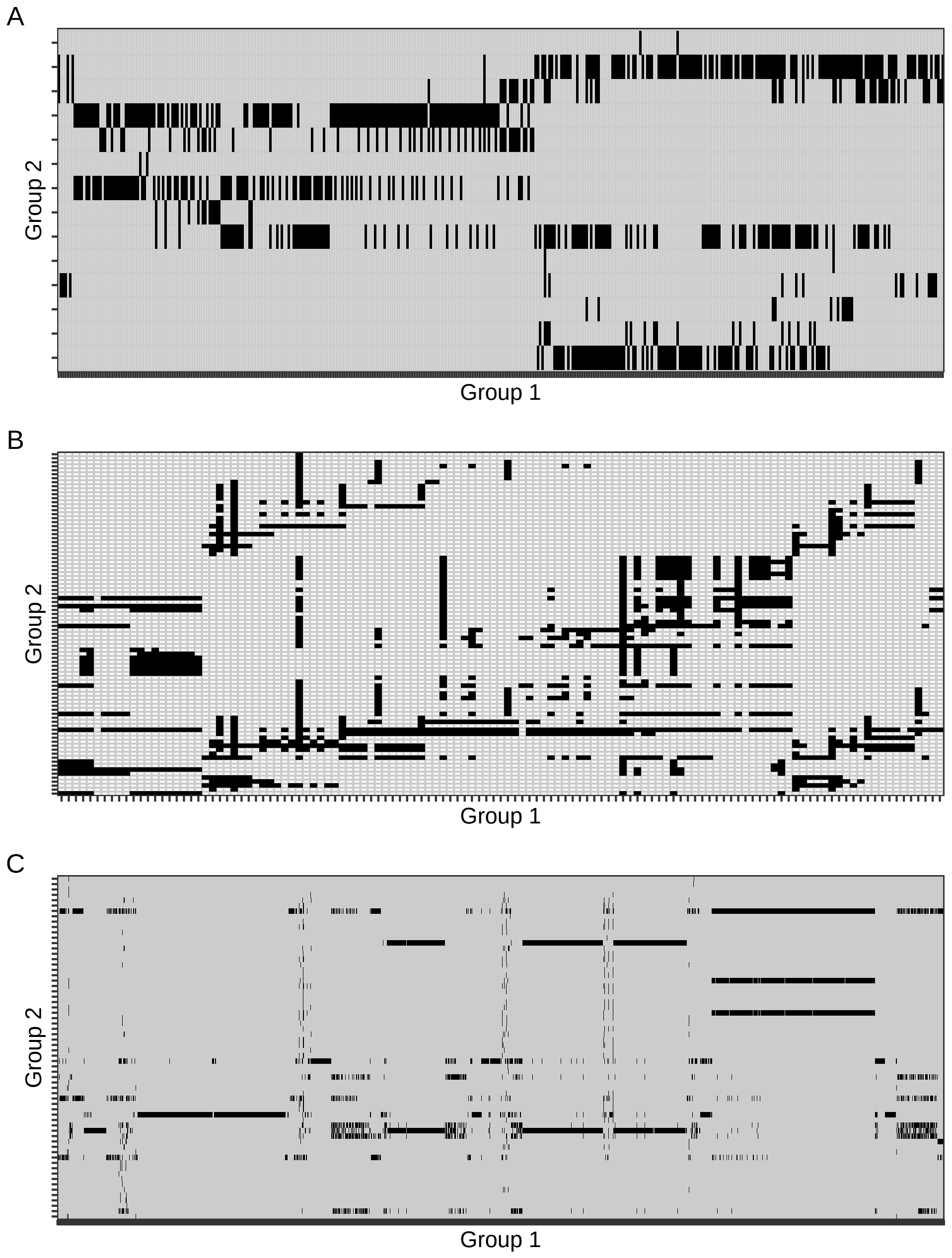

Fig. S2 Crossing matrices of the datasets indicating the sparsity/completeness of the factorial. Black tiles represent realized crosses. Tick marks on both axes represent the parents from the respective group. **A**: Ra1, 14 × 381; **B**: Mz1, 86 × 123; **C**: Mz2, 64 × 2100

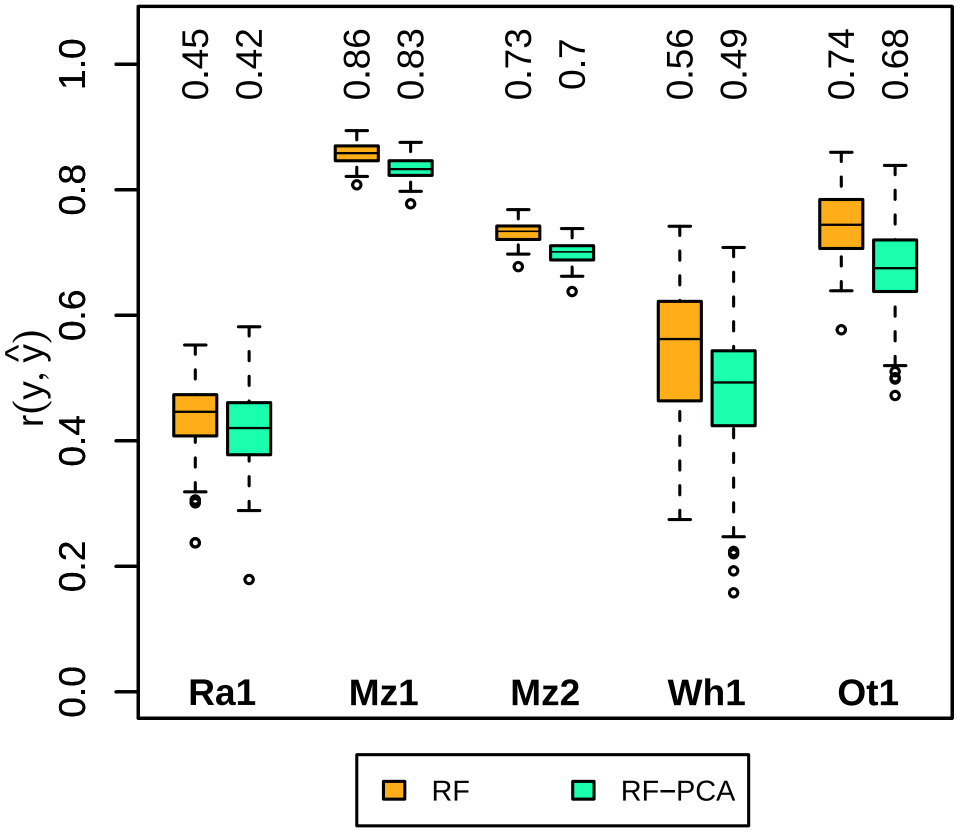

Fig. S3 Prediction accuracy of all cross-validation runs for all datasets, comparing RF based on the full SNPs and RF based on principal components of a PCA. Median prediction accuracy displayed above boxplots. RF on SNPs corresponds to the results presented in the main body of the study. When principle components were used as inputs, all principle components were included.
